# Retinal Levels of Amyloid Beta Correlate with Cerebral Levels of Amyloid Beta in Young APPswe/PS1dE9 Transgenic Mice before Onset of Alzheimer's Disease

**DOI:** 10.1155/2020/1574816

**Published:** 2020-09-24

**Authors:** Xi Mei, Mengxiang Yang, Lina Zhu, Qi Zhou, Xingxing Li, Zhongming Chen, Chenjun Zou

**Affiliations:** ^1^Kangning Hospital of Ningbo, Ningbo City, Zhejiang Province, China; ^2^Ningbo University, Ningbo City, Zhejiang Province, China; ^3^Weifang Medical University, Weifang City, Shandong Province, China

## Abstract

**Objectives:**

Retina abnormalities are related to cognitive disorders in patients with Alzheimer's disease (AD). Retinal amyloid beta (A*β*) can be labeled by curcumin. We measured A*β* content in the cerebrum and retina of APPswe/PS1dE9 (APP) transgenic mice with early age to investigate the correlation between cerebrum and retina.

**Methods:**

APP mice and age-matched wild-type mice were investigated every month from age 2 months to 6 months to assess changes in A*β* content in the retina and cerebrum. At the beginning of each month, mice were fed a curcumin diet (50 mg/kg/day) for 7 consecutive days. The A*β* levels in the retina and cerebrum were measured by ELISAs. Correlations were identified between retinal and cerebral A*β* contents using Pearson's correlation.

**Results:**

In the absence of curcumin, there was a significant correlation between A*β* contents in the retina and cerebrum of APP mice (*r* = 0.7291, *P* = 0.0014). With increasing age, A*β*-mediated degenerative change in the cerebrum (*P* < 0.001 in 5 months) and retina (*P* < 0.01 in 5 months) increased significantly. The inhibitory effect of curcumin on the A*β* level was significant in the cerebrum (*P* < 0.001) and retina (*P* < 0.01) of older APP mice in the early stage of life.

**Conclusion:**

We observed a significant correlation between the A*β* content in the retina and A*β* content in the cerebrum of APP mice. Our data suggest an appropriate time to measure retinal A*β*. Although curcumin can label A*β* in the retina, it also suppresses A*β* levels and weakens the degree of correlation between A*β* in cerebrum and retina tissues.

## 1. Introduction

Early diagnosis of Alzheimer's disease (AD) is essential for treatment [1]. Most diagnostic methods for AD are based on clinical symptoms [2]. Commonly, people in the presymptomatic stage of AD have no clinical symptoms (including impairment in episodic memory).

“Amyloid beta” (A*β*), which denotes peptides of 36-43 amino acids, is a major pathologic hallmark in the central nervous system (CNS) of patients with AD [3, 4]. Amyloidal precursor protein (APP) is first cleaved by the enzymes *β*-secretase and *γ*-secretase and then released into the space between cells [5]. Due to the different cleavage sites of *γ*-secretase, A*β* lengths are different. Soluble A*β* oligomers are more toxic than deposited plaques [6]. Although A*β* plaques are found in the brains of many elderly people without AD, they might be denoting a presymptomatic stage of AD.

Auxiliary diagnoses involve the examination of cerebrospinal fluid and positron emission tomography (PET) of A*β* plaques [7, 8]. The examination of cerebrospinal fluid A*β* is an invasive method. High cost of A*β*-PET limits its use in early diagnosis. Recent studies demonstrate the ability to detect CNS A*β* deposition via the use of plasma assessment of A*β* species [9, 10]. Due to the blood-brain barrier, plasma amyloid beta levels cannot reflect the real condition in the brain. Plasma concentrations of A*β*40 and A*β*42 have been shown to increase with age and in early AD but may decrease with advancing AD. However, no significant differences in plasma A*β* concentrations have been reported between individuals with and without AD [11].

However, AD is a disease of the CNS, and A*β* may distribute in all parts of nervous tissue, including the cerebrum and retina [12]. Ocular amyloidal imaging has been used to diagnose AD and monitor, noninvasively, AD progression [13].

Although the identifiable difference in the retinal structure between AD patients and healthy people is not straightforward, and misdiagnoses can occur [14, 15], retinal A*β* plaques which can be labeled with curcumin may improve diagnostic accuracy [16, 17]. This method merits further study and development into a new diagnostic measurement. Moreover, curcumin is a safe, nontoxic lipophilic agent with antioxidant and anti-inflammatory properties [4]. Apart from being a valuable labeling agent, curcumin may also play an important part in the AD treatment without eliciting side effects [18, 19].

The biological basis of curcumin-labeled examination of the retina is good coherence between the cerebrum and retina. Retinal A*β* plaques can reflect A*β* plaques in the cerebrum. When is the best time to early detect A*β* and diagnose AD through the retina? What is the consistency between the cerebral A*β* and the retinal A*β*? In this brief research report, we elucidated A*β* content and its coherence in the cerebrum and retina of mice with early-age before the onset of AD.

## 2. Materials and Methods

### 2.1. Ethical Approval of the Study Protocol

The study protocol was approved by the Animal Care and Use Committee of the Medical School of Ningbo University (Ningbo, China). Animal experiments were undertaken according to the *Guide for the Care and Use of Laboratory Animals* (National Institutes of Health (Bethesda, MD, USA) publication number 80-23, revised 1996).

### 2.2. Animals

APPswe/PS1dE9 transgenic mice (APP) and age-matched wild type (WT) mice were provided by the Model Animal Research Center of Nanjing University (Nanjing, China). To exclude the effect of sex on results, only male mice were used. Animals were housed in cages in a room maintained at 22 ± 2°C and 60 ± 5% relative humidity under a 12 h light–dark cycle (lights on at 6 : 00 am). Water and food were freely available in their cages. Animal experiments were conducted outside of their housing area in a separate room.

### 2.3. Experimental Procedures

According to previous studies, amyloid-beta plaques in CNS of AD mice are markedly formed at 6 months of age [20]. Curcumin was administered to 2-, 3-, 4-, 5-, and 6-month old APP and WT mice for 7 consecutive days using the intragastric (i.g.) administration route.

Mice at the age of each month were divided into 4 experimental groups (*n* ≥ 3 mice/group): (1) APP mice treated with curcumin (50 mg/kg/day) dissolved in phosphate-buffered saline (PBS, 0.1 mg/g), *n* = 19; (2) APP mice treated with the same volume of only PBS, *n* = 17; (3) WT mice treated with curcumin (50 mg/kg/day) dissolved in phosphate-buffered saline (PBS, 0.1 mg/g), *n* = 18; and (4) WT mice treated with the same volume of only PBS, *n* = 17.

The dose of curcumin used in this study was chosen according to previous animal studies and clinical trials [16, 21, 22]. According to previous studies, at high dosages, curcumin might prevent short-term recognition but not spatial memory. No signs of neurogenesis were evident, but reduced neuroinflammation was observed. The dose of the intragastric (i.g.) administration route has been shown to reduce the risk of vascular inflammation in the brain of AD subjects [19, 23].

Mice were sacrificed by neck amputation after the final administration. Then, the retina and cerebrums were isolated. Tissue samples were homogenized in RIPA Buffer (Beijing Solarbio Science & Technology, Beijing, China) at 1 : 10 (*g*/*v*) with 1% phenylmethylsulfonyl fluoride (Beijing Solarbio Science & Technology). Supernatant proteins were extracted after centrifugation (13,000 rpm or 20 min at 4°C). For each sample, 150 *μ*L of extracted protein was used for detection. The A*β* concentration was quantified using a mouse total A*β* ELISA kit (Shanghai Yuanye BioTechnology, Shanghai, China) according to manufacturer protocols.

### 2.4. Materials

Curcumin (pure curcumin ≥ 80%, Hushi, Shanghai, China) was dissolved by phosphate-buffered saline (0.1 M Na_2_HPO_4_, 0.1 M KH_2_PO_4_, 0.1 M KCl, and 0.1 M NaCl, pH 7.4).

### 2.5. Enzyme-Linked Immune Sorbent Assay (ELISA)

The A*β* level in the brain and retina was measured by ELISAs. Absorbance at 450 nm (at a reference wavelength of 690 nm) was measured by an absorbance reader (Sunrise™; Tecan, Geneva, Switzerland). The absorbance value was transformed into a concentration value by reading the absorbance of pure samples on a standard curve.

### 2.6. A*β* Immunohistochemistry

Briefly, after anesthetized, mice were perfused with saline until the limbs and the liver turn white, and then perfused with 4% paraformaldehyde until the tail became stiff. The brain tissue was dissected and incubated with 4% paraformaldehyde for 1 day. After being washed with PBS, the tissue was put in a centrifuge tube containing 30% sucrose solution until the brain tissue sunk to the bottom. A cryostat was used to cut the brain tissue into 25 *μ*m thick brains. The sections were incubated in 1% BSA for 1 h and then incubated with *β*-amyloid antibody (1 : 500, Cell Signaling Technology) at 4°C overnight. The sections were washed 3 times with PBS, rinsed, and incubated with the secondary antibody at 37°C for 1 h. After staining with 4′,6-diamidino-2-phenylindole (DAPI) for 1 min, the sections were washed 3 times with PBS and imaged using a confocal fluorescence microscope.

### 2.7. Statistical Analyses

Data are the mean ± standard error (SE). Prism v7.0 (GraphPad, San Diego, CA, USA) was employed for statistical analyses. Differences among multiple mean ± SE values were assessed by one-way and two-way ANOVA, followed by Bonferroni's *post hoc* test. Differences between two mean ± SE values were assessed by the unpaired *t*-test. The correlation between A*β* content in the retina and cerebrum in each group was tested using Pearson's correlation. *P* < 0.05 was considered significant.

## 3. Results

### 3.1. A*β* Accumulation with Increasing Age in the Cerebrum of Mice

Several works have shown abundant A*β* plaques in the brain of APP transgenic mice older than 6 months [20]. Mice in the present study were aged 2-6 months, so A*β* plaques have not yet formed in the brain. Hence, we measured the A*β* concentration in the brain of each mouse using ELISAs. WT mice given or not given curcumin are represented as WT_C_ and WT_N_, respectively. APP mice given or not given curcumin are represented as APP_C_ and APP_N_, respectively.

**Figure 1:**
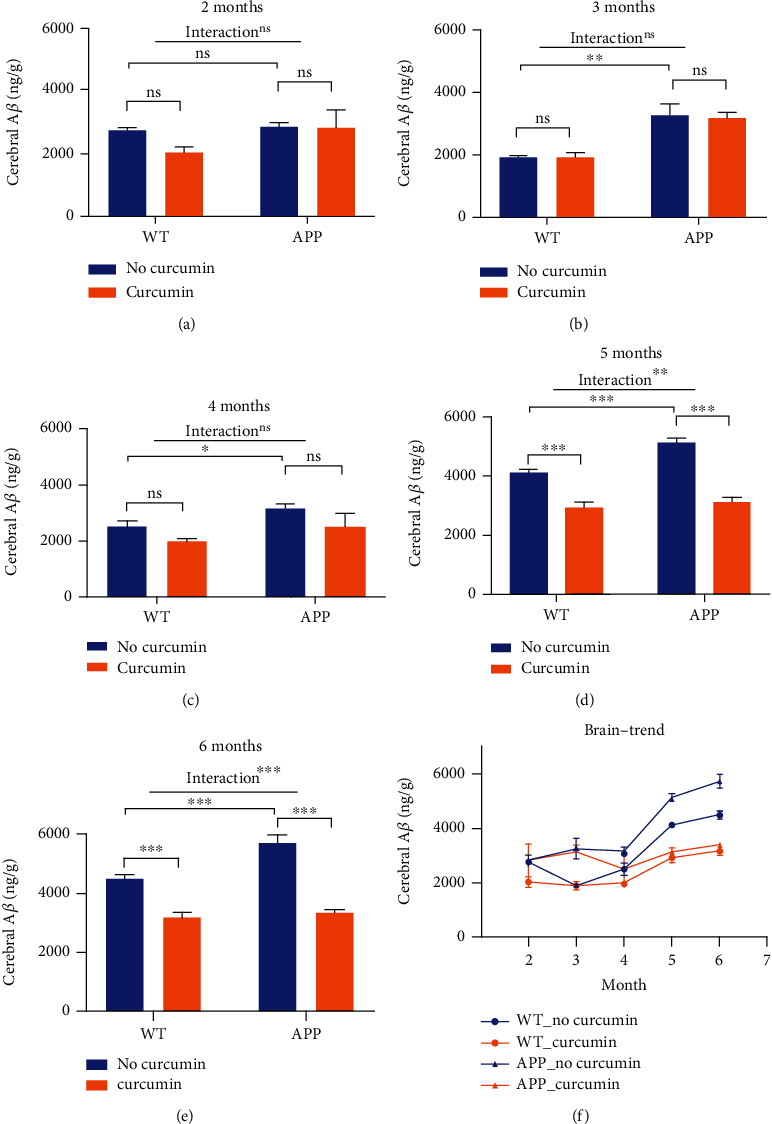
Effects of curcumin on the A*β* level in the cerebrum. Abbreviations: APP: APPswe/PS1dE9; WT: wild type; APP_C_: APP mice given curcumin (*n* = 19); APP_N_: APP mice not given curcumin (*n* = 17); WT_C_: WT mice given curcumin (*n* = 18); WT_N_: WT mice not given curcumin (*n* = 17); ^∗^*P* < 0.05; ^∗∗^*P* < 0.01; ^∗∗∗^*P* < 0.001.

Results revealed that A*β* was present in all four groups (WT_C_, WT_N_, APP_C_, APP_N_).  Figure 1 shows the change in the A*β* level in the four groups with increasing age, as well as the effects of curcumin on the A*β* level in mouse brains. A*β* content was significantly higher in the APP_N_ group compared with that in the WT_N_ group (*P* < 0.05 from 3 month to 6 month). A two-way ANOVA (genotype × treatment) revealed a significant effect of genotype (*F*(1, 8) = 100.5, *P* < 0.001) from 3 months to 6 months.

**Figure 2:**
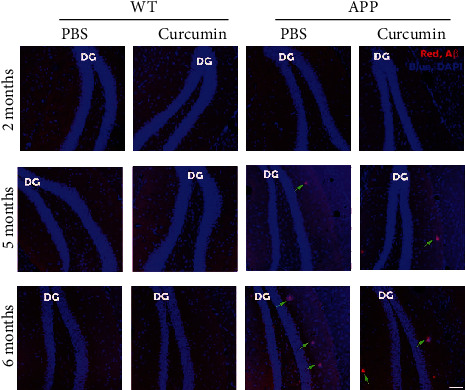
A*β* immunohistochemistry of hippocampus (red, A*β*; blue, DAPI). WT control brain exhibited no plaques in each group of age, while plaques of APP mice aged 5 and 6 months began to accumulate (green arrows). Abbreviations: APP: APPswe/PS1dE9; WT: wild type. Scale bar = 100 *μ*m.

From 5 months of age, the difference in the A*β* level between the curcumin administration group and no curcumin administration increased. The inhibitory effect of curcumin on the A*β* level was significant in the brain in 5 months, with 38.82% suppression by curcumin (APP_C_*vs*. APP_N_, *P* < 0.001). Images of Figure 2 show a progressive increase in plaque load of APP mice. The A*β* plaques began to accumulate from 5 months of age in tissue level.

### 3.2. Retinal Content of A*β* in Mice Aged 2-6 Months

**Figure 3:**
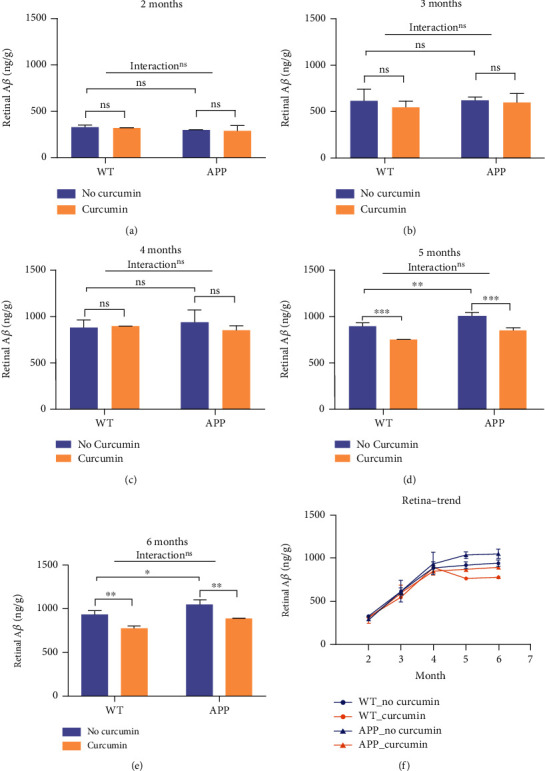
Effects of curcumin on the A*β* level in the retina. Abbreviations: APP: APPswe/PS1dE9; WT: wild type; APP_C_: APP mice given curcumin (*n* = 19); APP_N_: APP mice not given curcumin (*n* = 17); WT_C_: WT mice given curcumin (*n* = 18); WT_N_: WT mice not given curcumin (*n* = 17); ^∗^*P* < 0.05; ^∗∗^*P* < 0.01.

Different from the cerebrum, there was no significant difference in the A*β* level in the retinas between the APP_N_ group and the WT_N_ group until 5 months in Figure 3. At 5 months of age, the difference between APP_C_ and APP_N_ was significant, with 15.96% inhibition by curcumin being recorded (*P* < 0.01).

### 3.3. Deposition Trend of A*β* in the Cerebrum and Retina

There was an age-related increase in the A*β* content, as shown in Figures 1(f) and 2(f). A*β* accumulation in the brain was different from 2 months to 6 months of age. At 5 months of age, curcumin had a significant effect on the A*β* content. The retinal content of A*β* in mice aged 2-4 months was not sensitive to the effects of curcumin.

**Figure 4:**
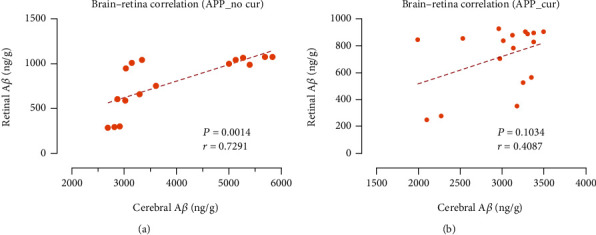
Correlations of the A*β* level in the cerebrum and retina of APP transgenic mice with (b) and without (a) administration of curcumin. Abbreviations: APP: APPswe/PS1dE9.

Correlation analyses between the A*β* level in the retina and that in the cerebrum are shown in Figure 4. Using combined data from APP mice of all ages, the A*β* level in the retina was correlated positively with the A*β* level in the cerebrum. Without curcumin administration, the A*β* level in the retina was correlated significantly with the A*β* level in the cerebrum (*r* = 0.7291, *P* < 0.01). Coherence between the brain and retina was established gradually with increasing age.

## 4. Discussion

In 2009, Perez and coworkers suggested that A*β* deposition within the retina can contribute to retinal dysfunction [24]. Many studies have focused on finding the best time to detect A*β* deposition in the eye because it could aid the early diagnosis of AD in humans [25, 26]. Aside from A*β* deposition, studies have focused on retinal function (e.g., light reflection) in young mice with AD [12]. However, A*β*-mediated degenerative change in the cerebrum and retina before the onset of AD has not been studied.

APPswe/PS1dE9 transgenic mice display early onset of A*β* deposition in the CNS [27, 28]. Hence, APPswe/PS1dE9 transgenic mice could be an ideal model to study the A*β*-related pathogenic effects on the nervous system in early-stage AD and even stage before the onset of AD.

Using APPswe/PS1dE9 transgenic mice, our study reported that in early age just before AD onset, there was a correlation between amyloid-beta levels of cerebrum and retina. Researchers have found A*β* plaques on the retina with the aid of the fluorescence effect of curcumin [17]. We also investigated the effect of curcumin on A*β* levels in CNS. Oral administration of curcumin can not only label the amyloid-beta plaques in the retina which may be a window of AD noninvasive diagnosis as Koronyo et al.'s study but also suppress the amyloid-beta level and its correlation of cerebrum and retina.

Time is an important factor in AD pathology. Notably, the accumulation of amyloid-beta increased over time. At 5 months old, the A*β* content became significantly different between mice of APP and WT genotype. The difference between A*β* levels of curcumin and no curcumin groups was higher from 5 months to 6 months. Before 4 months, cerebral levels of amyloid-beta were more affected by genotype than curcumin.

In the cerebrum of young mice, the difference in the A*β* level between APP and WT came earlier than that in the retina. This finding may be because A*β* accumulates in the hippocampal region at the beginning of life and then diffuses to frontal and temporal cortices and other parts of the CNS [1]. With increasing age, the A*β* level in the retina of APPswe/PS1dE9 transgenic mice increased. This appears to resemble a process of age-related decrease in the number of synapses in retinal layers as a result of A*β* accumulation [29]. A*β* accumulation is the upstream event and can be indirectly reflected by age-related increase in presynaptic and a decrease in postsynaptic retinal proteins in retinal plexiform layers [3, 29]. These phenotypes were similar to the brain. At this time (especially in 5-month-old mice), the amyloidal pathogenesis of AD compared with that in the normal retina could be distinguished. This may be the best time to detect amyloidal pathogenesis from retina for early diagnosis of AD.

Several research teams have used curcumin to label A*β* in the retina and brain [30]. Curcumin can also suppress A*β* accumulation. In our study, curcumin decreased the A*β* level in the brain and retina of APPswe/PS1dE9 transgenic mice in the early stage of life, but it had a more potent inhibitory effect in older mice. Feeding demethylcurcumin or bisdemethylcurcumin to APPswe/PS1dE9 double-transgenic mice can upregulate the NEP expression in the brain and reduce A*β* accumulation in the hippocampus and cortices of mice at 4.5-5.5 months of age [31].

In the absence of curcumin, the trends of A*β* accumulation in APPswe/PS1dE9 transgenic mice and WT mice were inconsistent and continued to increase with increasing age. However, in APPswe/PS1dE9 transgenic mice, there was a significant correlation between the A*β* content in the retina and brain. In addition to A*β*, hyperphosphorylation of tau proteins disrupts the retinal structure and may contribute to visual deficits seen in APPswe/PS1dE9 transgenic mice [24, 32].

Besides the retina, other ocular changes occur in AD patients and animals: altered pupil flash response, A*β* aggregation in the lens, and abnormal pattern electroretinograms [33–36]. Hence, even though most AD-related disease occurs in the brain, AD can also affect the eye. The retina shares many features with the brain (embryological origin, anatomic (e.g., microvascular bed) and physiologic (e.g., blood-tissue barrier) characteristics) [14], so the relationship between the brain and retina merits further study.

Ocular imaging of A*β* in AD patients could facilitate noninvasive monitoring [13]. In humans, ophthalmic imaging methods are used to assess neurodegenerative disorders, such as AD and Parkinson's disease [15]. Presently and in the future, the relationship between the degree of cognitive impairment and retinal abnormality merits further the study (Table 1).

A*β* is also observed in virtually all people with Down syndrome aged > 40 years and leads to a clinical diagnosis of dementia [37]. Increased retinal nerve fiber layer (RNFL) thinning in adults with Down syndrome may represent early AD-related changes. Studies in other neurodegenerative diseases, such as multiple sclerosis and cognitive decline, have also suggested that a thinner RNFL may be a preclinical observation of dementia [38, 39].

As a potential contrast agent of AD retinal diagnosis, pharmacokinetics of curcumin in wild type and APP mice should be further explored. A previous study investigated a magnetic resonance imaging contrast agent [40]. They reported that no significant differences were observed in the plasma or brain kinetics of wild type and APP mice. Similar to curcumin, this contrast agent was previously shown to cross the blood-brain barrier and bind to amyloid plaques in the brain of AD transgenic mouse.

With the advent of advanced imaging technologies and A*β* biomarkers for clinical use, it is now possible to identify the effects of A*β* accumulation through noninvasive imaging of ocular structures in live patients. Optical coherence tomography angiography (OCTA) was used in clinical trials of AD detection, but only revealed the structure of biological tissues, such as RNFL thickness and vessel density [39]. Therefore, a detection method based on pathological biomarkers is urgently needed. In the future, AD progression could be quantified by measurement of the retinal A*β* level. One limitation of the proposed method is that although curcumin can label A*β* in the retina, it also suppresses A*β* levels and weakens the degree of correlation between A*β* in cerebrum and retina tissues.

## 5. Conclusions

We observed a significant correlation between the A*β* content in the retina and A*β* content in the brain of young APP mice before the onset of AD. Our data provide a biological basis for supporting noninvasive detection of AD in the eye and also suggest a time to detect retinal A*β*. Although curcumin can label the A*β*, it can also suppress the A*β* level and weaken the degree of correlation.

## Figures and Tables

**Table 1:** RNFL thickness inpatients of different ages with neurodegenerative disease.

Age (years)	*N*	Female : male	Subjects	Cognitive evaluation	RNFL thickness	Reference
35.80 ± 9.48	49	26 : 23	Down syndrome	Temporal cortex MRI and PET	*r* = 0.592^∗^	[37]
43.4 ± 12.0	66	48 : 18	Multiple sclerosis	MRI	*B* = 1.26^∗∗^	[38]
56.0 (55.9 to 56.1)	32038	17172 : 14866	Cognitive decline	Cognitive function tests	OR = 2.08^∗∗∗^	[39]
65.36 ± 5.55	56	35 : 21	Preclinical AD	PET	-0.228^∗^	[41]
73.5 ± 6.0	63	32 : 31	AD	PET/CT	AUC = 0.652^∗^	[42]

^∗^ *P* < 0.05; ^∗∗^*P* < 0.01; ^∗∗∗^*P* < 0.001; AD: Alzheimer's disease; MRI: magnetic resonance imaging; PET: positron emission tomography; CT: computed tomography; OR: odds ratio; AUC: area under the receiver operating characteristic curves (to assess the ability of RNFL thicknesses to discriminate AD cases from healthy people).

## Data Availability

All data are included in the manuscript. However, the raw data used and/or analyzed in the present study are available from the corresponding author on reasonable request.
